# Supplementary material for: Genomic plasticity and rapid host switching can promote the evolution of generalism: a case study in the zoonotic pathogen Campylobacter
Source: Sci Rep. 2017 Aug 29;7:9650. doi: 10.1038/s41598-017-09483-9 (PMC5575054; doi:10.1038/s41598-017-09483-9)
Supplement: Supplementary file 1 — Supplementary material [file 41598_2017_9483_MOESM1_ESM.pdf]

Supplementary material for:

Genomic plasticity and rapid host switching can promote the evolution of generalism: a case study in the zoonotic pathogen *Campylobacter*

Dan J. Woodcock[1], Peter Krusche[1], Norval J. C. Strachan[2], Ken J. Forbes[3], Frederick M. Cohan[4], Guillaume Méric[5], Samuel K. Sheppard[5,6]*

[1]Warwick Systems Biology Centre, Coventry House, University of Warwick, Coventry, CV47AL, UK; [2]School of Biological Sciences, University of Aberdeen, Cruickshank Building. St Machar Drive, Aberdeen, AB24 3UU, UK; [3]School of Medicine and Dentistry, The University of Aberdeen, Foresterhill, Aberdeen, AB25 2ZD, UK; [4]Department of Biology, Wesleyan University, Middletown, CT 06459-0170, USA; [5]The Milner Centre for Evolution, Department of Biology and Biochemistry, University of Bath, Claverton Down, Bath. BA2 7AY, UK; [6]Department of Zoology, University of Oxford, South Parks Road, Oxford, OX1 3PS, UK.

* Corresponding author: s.k.sheppard@bath.ac.uk.

Figure S1.

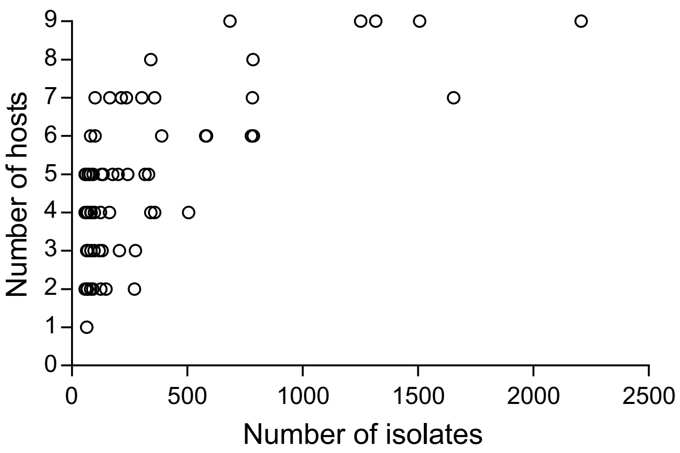

9
8
7
6
5
4
3
2
1
0
Number of hosts
0
500
1000
1500
2000
2500
Number of isolates

Table S1. *Campylobacter* genomes used in this study.

Identifier	Isolate name	Country of isolation	Year of isolation	Sequence type (ST)	Clonal complex (CC)	Host of isolation
4	CAMP45	UK	2005	45	ST-45 complex	chicken
13	CAMP61	UK	2006	61	ST-61 complex	cattle
14	CAMP2381	New Zealand	n/a	2381	n/a	wild bird
22	CAMP2488	UK	2001	257	ST-257 complex	chicken
26	NC 009839	UK	2007	267	ST-283 complex	n/a
27	NC 008787	n/a	n/a	604	ST-42 complex	n/a
28	NC 003912	n/a	n/a	354	ST-354 complex	n/a
29	NC_002163	n/a	n/a	43	ST-21 complex	clinical
30	CAMP1044	UK	2007	n/a	n/a	n/a
32	CampsClin11	UK	2005	11	ST-45 complex	clinical
34	CampsClin262	UK	2005	262	ST-21 complex	clinical
36	CampsClin266	UK	2006	266	ST-21 complex	clinical
37	CampsClin883	UK	2006	883	ST-21 complex	clinical
39	chick2219	UK	2005	2219	ST-45 complex	chicken
40	chicka21	UK	2006	21	ST-21 complex	chicken
42	cow42	UK	2006	42	ST-42 complex	cattle
43	chick2253	UK	2006	n/a	n/a	chicken
45	chick594	UK	2006	583	ST-45 complex	chicken
48	cow206	UK	2006	206	ST-206 complex	cattle
49	cow38	UK	2006	38	ST-48 complex	cattle
52	cow334	UK	2006	334	ST-45 complex	cattle
54	chick267	UK	2005	267	ST-283 complex	chicken
55	CampsClin230	UK	2006	230	ST-45 complex	clinical
56	cowa45	UK	2006	45	ST-45 complex	cattle
57	chick2213	UK	2005	334	ST-45 complex	chicken
59	cow518	UK	2006	21	ST-21 complex	cattle
60	CampsClin53	UK	2005	53	ST-21 complex	clinical
62	cowa21	UK	2006	21	ST-21 complex	cattle
63	chickc21	UK	2006	21	ST-21 complex	chicken
64	chick25	UK	2006	814	ST-661 complex	chicken
65	chick104	UK	2006	104	ST-21 complex	chicken
66	chick353	UK	2009	353	ST-353 complex	chicken
67	chickb354	UK	2009	354	ST-354 complex	chicken
68	chick573	UK	2009	573	ST-573 complex	chicken
69	chick2568	UK	2009	2568	ST-661 complex	chicken
70	chickc45	UK	2009	45	ST-45 complex	chicken
71	chick19	UK	2009	50	ST-21 complex	chicken
72	chick50	UK	2009	50	ST-21 complex	chicken
73	chick53	UK	2009	53	ST-21 complex	chicken
74	chick262	UK	2009	262	ST-21 complex	chicken

75	chick266	UK	2009	266	ST-21 complex	chicken
77	chick1086	UK	2009	50	ST-21 complex	chicken
78	chick1360	UK	2009	50	ST-21 complex	chicken
79	chick11	UK	2009	11	ST-45 complex	chicken
80	chick137	UK	2009	2030	ST-257 complex	chicken
81	chick1003	UK	2009	1003	ST-45 complex	chicken
82	chick2048	UK	2009	45	ST-45 complex	chicken
83	chick2197	UK	2009	354	ST-354 complex	chicken
84	chick2223	UK	2009	45	ST-45 complex	chicken
85	cow3583	UK	2003	3583	ST-42 complex	cattle
86	cow618	UK	2003	61	ST-61 complex	cattle
87	cow273	UK	2003	273	ST-206 complex	cattle
88	cow270	UK	2003	270	ST-403 complex	cattle
89	cowb21	UK	2003	21	ST-21 complex	cattle
90	cowb45	UK	2003	45	ST-45 complex	cattle
91	cowc45	UK	2003	45	ST-45 complex	cattle
92	cowd45	UK	2003	45	ST-45 complex	cattle
94	cow104	UK	2003	104	ST-21 complex	cattle
96	cow3189	UK	2003	n/a	n/a	cattle
97	cow3201	UK	2003	19	ST-21 complex	cattle
99	cow3205	UK	2003	206	ST-206 complex	cattle
100	cow137	UK	2003	137	ST-45 complex	cattle
102	cow583	UK	2003	583	ST-45 complex	cattle
103	cow3207	UK	2003	334	ST-45 complex	cattle
104	cow3214	UK	2003	45	ST-45 complex	cattle
105	chick354	UK	2004	257	ST-257 complex	chicken
106	chick51	UK	2005	51	ST-443 complex	chicken
107	chick1079	UK	2004	1079	ST-573 complex	chicken
108	chick574	UK	2004	574	ST-574 complex	chicken
109	chick814	UK	2004	814	ST-661 complex	chicken
110	chickb21	UK	2003	21	ST-21 complex	chicken
111	chickb45	UK	2004	45	ST-45 complex	chicken
112	chickd45	UK	2004	45	ST-45 complex	chicken
113	chick883	UK	2004	883	ST-21 complex	chicken
114	chick230	UK	2004	230	ST-45 complex	chicken
116	CampsClin21	UK	2005	n/a	n/a	clinical
117	OxClina21	UK	2003	21	ST-21 complex	clinical
119	OxClina45	UK	2003	45	ST-45 complex	clinical
122	starling177	UK	n/a	177	ST-177 complex	wild bird
124	starling45	UK	n/a	45	ST-45 complex	wild bird
125	starling1020	UK	n/a	1020	ST-682 complex	wild bird
126	goose1033	UK	n/a	1033	ST-1034 complex	wild bird
127	goose702	UK	n/a	n/a	n/a	wild bird
128	goose137	UK	n/a	137	ST-45 complex	wild bird

129	goose696	UK	n/a	696	ST-1332 complex	wild bird
130	duck702	UK	n/a	702	ST-702 complex	wild bird
131	duck45	UK	n/a	45	ST-45 complex	wild bird
172	Cj129-258	USA	n/a	459	ST-42 complex	cattle
173	Cj51494	USA	n/a	4834	ST-353 complex	chicken
174	CjLMG23216	Belgium	n/a	4835	n/a	chicken
175	CjLMG23218	Belgium	n/a	48	ST-48 complex	chicken
176	CjLMG23223	Belgium	n/a	791	n/a	chicken
177	CjLMG23263	Bosnia	n/a	3504	ST-446 complex	chicken
178	Cj60004	USA	n/a	4836	n/a	chicken
179	CjLMG23264	Slovenia	n/a	46	ST-206 complex	clinical
180	CjLMG23269	Belgium	n/a	4837	ST-353 complex	chicken
181	Cj55037	USA	n/a	45	ST-45 complex	chicken
182	CjLMG9879	Canada	n/a	47	ST-21 complex	clinical
183	Cj86605	USA	n/a	4840	ST-48 complex	chicken
184	CjLMG23357	Netherlands	n/a	4883	ST-1275 complex	n/a
185	CjATCC33560T	Belgium	n/a	403	ST-403 complex	cattle
186	CjLMG9081	SouthAmerica	n/a	52	ST-52 complex	clinical
187	Cj53161	USA	n/a	4838	ST-353 complex	chicken
188	CjLMG9217	Belgium	n/a	443	ST-443 complex	clinical
189	Cj2008-1025	France	n/a	50	ST-21 complex	clinical
190	Cj2008-894	France	n/a	1962	n/a	clinical
191	Cj2008-872	France	n/a	61	ST-61 complex	clinical
192	Cj2008-988	France	n/a	572	ST-206 complex	clinical
193	Cj1997-1	USA	n/a	658	ST-658 complex	clinical
194	Cj2008-979	France	n/a	2274	n/a	clinical
195	Cj2008-831	France	n/a	50	ST-21 complex	clinical
196	Cj1997-4	USA	n/a	475	ST-48 complex	clinical
197	Cj1997-7	USA	n/a	61	ST-61 complex	clinical
198	Cj1997-10	USA	n/a	4839	n/a	clinical
199	Cj1997-11	USA	n/a	22	ST-22 complex	clinical
200	Cj1997-14	USA	n/a	5159	ST-353 complex	clinical
201	Cj51037	USA	n/a	939	ST-353 complex	chicken
202	Cj110-21	USA	n/a	982	ST-21 complex	cattle
203	Cj87330	USA	n/a	50	ST-21 complex	chicken
204	Cj87459	USA	n/a	452	ST-353 complex	chicken
205	Cj140-16	USA	n/a	5161	ST-61 complex	cattle
206	Cj1213	USA	n/a	132	ST-508 complex	cattle
207	CjATCC43432	Canada	n/a	122	ST-206 complex	clinical
208	Cj1798	USA	n/a	61	ST-61 complex	cattle
209	Cj1854	USA	n/a	922	n/a	cattle
210	Cj1893	USA	n/a	38	ST-48 complex	cattle
211	Cj1928	USA	n/a	806	ST-21 complex	cattle
212	CjLMG9872	Sweden	n/a	677	ST-677 complex	clinical

213	Cj23210	Belgium	n/a	380	n/a	chicken
214	CjLMG23211	Belgium	n/a	220	ST-179 complex	chicken

Table S2. Model input genes.

Locus tag	Gene name	Genomic location [a]	COG [b]	Predicted function	COG code [b]	COG description [b]
Putative niche-specifying genes						
cj0007	*gltB*	8144..12634	COG0069	glutamate synthase large subunit	E	Amino acid transport and metabolism genes
cj0019c	-	23665..25443	COG0840	putative MCP-domain signal transduction protein	T	Signal transduction mechanisms genes
cj0644	-	606050..606865	COG0084	putative TatD-related deoxyribonuclease protein	L	Replication, recombination and repair
cj0665c	*argG*	621392..622612	COG0137	argininosuccinate synthase	E	Amino acid transport and metabolism genes
cj0671	*dcuB*	625552..626976	COG2704	anaerobic C4-dicarboxylate transporter	R	General function prediction only
cj0917c	*cstA*	852359..854470	COG1966	putative integral membrane protein	T	Signal transduction mechanisms genes
cj1259	*porA*	1189121..1190395	-	major outer membrane protein	S	Function unknown
cj1342c	*maf7*	1274694..1275935	-	motility accessory factor	S	Function unknown
cj1346c	*dxr*	1279921..1278851	COG743	1-deoxy-D-xylulose 5-phosphate reductoisomerase	I	Biosynthesis of cofactors, prosthetic groups, and carriers
cj1457c	*truD*	1395403..1394285	COG585	Predicted tRNA pseudouridine synthase D	S	Function unknown
MLST genes						
cj0087	*aspA*	96074..97480	COG1027E	aspartate ammonia-lyase	E	Amino acid transport and metabolism genes
cj0105	*uncA*	111488..112993	COG0056C	F0F1 ATP synthase subunit alpha	C	Energy production and conversion genes
cj0434	*pgm*	402285..403763	COG0696G	phosphoglyceromutase	G	Carbohydrate transport and metabolism genes
cj0699c	*glnA*	656901..658331	COG0174E	glutamine synthetase	E	Amino acid transport and metabolism genes
cj1682c	*gltA*	1603983..1605251	COG0372C	citrate synthase	C	Energy production and conversion genes

a. On the *C. jejuni* NCTC11168 reference genome

b. Cluster of Orthologous Groups, as obtained on: http://www.ncbi.nlm.nih.gov/COG/

Supplementary Material for

Genomic plasticity and rapid host switching promote the evolution of generalism in the zoonotic pathogen *Campylobacter*

Dan J. Woodcock[1], Peter Krusche[1], Norval J. C. Strachan[2], Ken J. Forbes[3], Frederick M. Cohan[4], Guillaume Méric[5] and Samuel K. Sheppard[5,6,7,*]

[1]Warwick Systems Biology Centre, University of Warwick, Coventry, CV4 7AL, UK
[2]School of Biological Sciences, The University of Aberdeen, Cruickshank Building, St Machar Drive, Aberdeen, AB24 3UU, UK
[3]School of Medicine and Dentistry, The University of Aberdeen, Foresterhill, Aberdeen, AB25 2ZD, UK
[4]Department of Biology, Wesleyan University, Middletown, CT 06459, USA
[5]Swansea University Medical School, Institute of Life Science, Singleton Park, Swansea, SA2 8PP, UK
[6]MRC CLIMB, Swansea University, Singleton Park, Swansea, SA2 8PP, UK
[7]Department of Zoology, University of Oxford, South Parks Road, Oxford, OX1 3PS, UK
[*]Corresponding author: `s.k.sheppard@swansea.ac.uk`

1 Modelling bacterial population dynamics *in silico*

Here we detail the model used throughout the main paper (MP) and discuss how the data was incorporated into the model and how simulations were performed.

1.1 Fitness function

The concept of fitness is fundamental to our approach and we model this using a fitness function, which allows us to quantify the fitness of the cell relative to its environment. Quantifying fitness has been the subject of much investigation, and a number of mathematical approaches have been developed which explore results and ramifications of applying various models. However, it is

beyond the scope of this study to evaluate the efficacy of the multitude of approaches to fitness quantification and so we opt for simple but valid approach to aid interpretation, namely the classical additive multilocus fitness model. This is an instance of the well known NK model [1] where no epistasis between loci is assumed, which is equivalent to setting $K = 0$.

In this model, each allele at each locus is assigned a value between 0 and 1 inclusive, $f(a_j) \in [0\ 1]$, which denotes the relative contribution the allele a at locus j gives to the overall fitness. The fitness of the cell, $F(C_i)$ is given by the mean of the individual fitness values of the constituent alleles of a cell

$$F(C_i) = \frac{1}{n}\sum_{j=1}^{n} f(a_j), \tag{1}$$

and cell i will survive and remain in the population with probability $F(C_i)$ per generation.

1.1.1 Selection of loci and determination of fitness

From our data set, we selected five loci that contained alleles which were most highly represented in the bacteria found in chicken but not in cow, and the five loci with alleles highly represented in the converse case and took these to be our 'niche-specifying genes'. Furthermore, we ensured that these alleles were in diverse positions upon the bacterial chromosome, which would break frequency dependence due to chromosomal proximity and confer more confidence in our assumption of no epistasis in our fitness function. It of course remains possible that we may have selected alleles where epistatic interactions occur due to functional interdependence but as this function would be likely to be related to the survival in a particular host, this is somewhat implicit in both the selection procedure and the model, and so should not greatly affect the outcome of the simulations.

Allelic fitnesses were determined by taking dividing the frequency of occurence of each allele n_{a_k} at a locus L_j by the frequency of occurrence of the most frequent allele n_{max} for each

locus L_j thus

$$f(a_k) = \frac{n_{a_k}}{n_{max}} \; \forall \; k \in L_j. \tag{2}$$

As such, the most frequent allele was assigned a fitness of 1, and the less frequent ones were given values relative to this. This was repeated for all ten loci. We used this method to construct fitnesses for the bacteria that were sampled from chickens and those that were sampled from cattle. Furthermore, we created a composite fitness function by elementwise addition of the allelic fitnesses of chicken and cattle, maximised at 1.

To these ten loci, we added five from the MLST schema [2] and the alleles at these loci were all given a fitness of 1 to reflect their neutral nature. These were used as a buffer so that even bacteria with low fitness had a chance of survival (unless highly mutated), but also so we could verify that the algorithm was working correctly and population mixing was occurring as expected as these alleles would become uniformly distributed in the populations, irrespective of fitness function.

1.2 Base Model

For the purposes of this study, we represent each cell by a sequence type that corresponds to the alleles found at each locus, selected as detailed above. The model incorporates five cellular processes: resource consumption, mutation, recombination, cell division and cell death. Cell death is further divided by its two causes, fitness and resource-related death. The replenishment of resources is also included in the model which confers an informal carrying capacity as the population will tend to an equilibrium where resources are being used as fast as they are being generated and the population cannot grow any further.

Mutation and recombination occur at the level of the individual locus and cell death and cell division occur at the ST (cell) level. With cell division, a copy of the cell that divides is added to the population, this occurs with rate b per generation. Mutation occurs with rate m per allele

per generation, and any allele that mutates is deemed to offer no selective advantage at all and is assigned a fitness of zero. Similarly, recombination occurs with rate r per allele per generation, and an allele that recombines is assigned the value of another allele randomly chosen from those at the same loci proportional to their frequency in the current population.

Resources are generated with a rate of g per generation and are utilised/consumed proportional to their abundance, where each of the $N_{pop} - N_{res}$ cells that are not already using resources in the current population have an equal chance of beginning to utilise a resources, with a rate of u^+ per generation for each resource R. The N_{res} cells in the population which are using a resource are immune to resource related death for a period of time, which elapses at rate u^- per generation. Cells which are unable to locate a resource have die at a rate of d per generation.

Including the fitness related death function from Section 1.1, the full set of processes governing this system can be represented as

$$\begin{aligned}
C_{i,j} &\xrightarrow{\quad m \quad} M, \\
C_{i,j} &\xrightarrow{\quad r \quad} C_{k,j},\ C_k \in \{C_i\} \\
C_i &\xrightarrow{\quad b \quad} 2C_i, \\
C_i &\xrightarrow{1-F(C_i)} \varnothing, \\
C_i^{(N)} &\xrightarrow{\quad d \quad} \varnothing, \\
C_i^{(N)} &\xrightarrow{u^+R(N_{pop}-N_{res})} C_i^{(U)}, \\
C_i^{(U)} &\xrightarrow{u^- N_{res}} C_i^{(N)}, \\
R &\xrightarrow{u^+R(N_{pop}-N_{res})} R-1, \\
\varnothing &\xrightarrow{\quad g \quad} R+1,
\end{aligned}$$

where C_i represents a cell with index i, and the superscript indicates whether cell i is currently using (U) or is not currently using (N) a resource. Where the superscript is omitted,

the resource status of the cell is irrelevant. $C_{i,j}$ denotes allele j of cell i. R are the number of resources, M is a mutant allele and $F(C_i)$ is the fitness of cell C_i.

1.2.1 Stochastic Model

We utilise a stochastic sampling algorithm in which each rate in the model is accorded a corresponding probability of occurence per time step. Performing such a simulation using the Gillespie algorithm would be computationally infeasible and so we use distributions to update the system in discrete steps. Most values were generated via a Binomial distribution ($B(n, p)$), which determines the number of successes given a number of trials n for a given probability of occurrence per trial p. This was used instead of the more common Poisson distribution for stochastic rate modelling as the number of trials was determined by population size which can vary in our model. As such, we split each generation into ten discrete time periods ($\Delta = 0.1$ generations) and evaluated the operations once per time period. This seemed to give the best tradeoff between accuracy and efficiency. We describe how each process is modelled stochastically at each time step, for a given population consisting of N_{pop} bacterial cells (of which the number using a resource was N_{res}), each consisting of K loci.

Mutation The number of mutations that will occur in a time step was calculated via a Binomial distribution, $n_m = B(N_{pop}, mK\Delta)$, and then we assigned the loci to mutate uniformly at random. Each mutation was given an identical identifier (allele number 0), which was assigned a fitness of 0.

Recombination The number of recombination events that will occur in a time step was calculated via a Binomial distribution $n_r = B(N_{pop}, rK\Delta)$, and then we assigned the donor alleles uniformly at random. We then determined recipient alleles at the same loci, again uniformly at random, and gave the recipient the allele number and fitness value of the donor.

Cell division The number of cell division events that will occur in a time step was calculated with a Binomial distribution $n_b = B(N_{pop}, b\Delta)$ and then we determined which cells were to divide at random. These cells were duplicated and added to the population.

Cell death (fitness-related) The fitness of each cell was calculated in turn and then we determined whether it survives by drawing a uniform random number q. If $q > 1 - F(C_i)$ then the cell dies and is removed from the population.

Cell death (resource-related) The number of cells that will die from those not using a resource was calculated using a Binomial distribution $n_d = B(N_{pop} - N_{res}, d\Delta)$, and we determined which cells to remove from the population uniformly at random.

Resource utilisation (cell) The number of cells that will start using a resource was calculated using a Binomial distribution $n_u = B(N_{pop} - N_{res}, uR\Delta)$ and this number of cells were selected from those not using a resource uniformly at random to become designated as using a resource.

Resource utilisation (resource) The utilisation of a resource also removes one of the resources from the population making it unable to be utilised. As each cell can only use exactly one resource at at time, when n_u is calculated using for the cell case above, this number is subtracted from the number of available resources, $R_{new} = R_{old} - n_u$.

Cessation of resource utilisation As the probability of cessation of resource utilisation is independent of the amount of time already spent using a resource (i.e. follows an exponential decay), we do not need to track the time spent using a resource. As such, the number of cells that will stop using a resource was calculated using a Binomial distribution $n_c = B(N_{res}, c\Delta)$ and this number of cells was selected from those using a resource uniformly at random and were

designated as not using a resource.

Resource generation As the number of resources is unbound, the generation of resources can be modelled with a Poisson distribution. As such the new number of resources is $R_{new} = R_{old} + Poiss(g\Delta)$.

1.2.2 Simulation

An initial population was randomly generated of 50 million cells with allele values appearing proportional to their frequency in the data set. The same initial population was used in every run.

The simulation began with the composite fitness function which was changed to the chicken fitness function after two hundred generations. The purpose of the composite fitness function is to allow alleles to align themselves in fit genotypes, and reach an equilibrium level with no bias toward either niche. In the first experiment, the fitness function was changed to the cow fitness function after a further 1000 generations, whereas in the second experiment it alternated between cattle and chicken every two hundred generations.

Parameter values used in the simulations are given in Table 1.2.2

2 Interplay between recombination and the two causes of cell death

Aside from imparting a carrying capacity, the resource abundance also creates an interesting interplay between fitness and resource related death in deciding the fate of the cell. When resources are high compared to the population, the predominant driver of cell death is fitness. Conversely, when the cell has approached its optimal genotype, the cause of death is predominantly resource-driven. Figure 1 shows the relative contribution of fitness and resources to the

Table 1: Parameters used in simulations

Process	Parameter	Value
Mutation	m	0.01
Recombination	r	varies
Cell division	b	$\log(2)^*$
Cell death (fitness)	$1 - F(C_i)$	varies
Cell death (resources)	d	0.9
Resource utilisation	u	1
Cessation of resource utilisation	c	$1^\dagger$
Resource generation	g	2000000

* $b = \log(2)$ ensures an unrestricted growth of approximately double per generation when dividing each generation up into multiple time steps.

† As cessation essentially follows an exponential decay, this parameter equal to 1 means the mean time of resource utilisation is one generation.

fate of a cell for a given population size in a simple simulation of one generation with a starting population size and fixed fitness.

This subtlety is important for understanding how the population dynamics affect the composition of the population, and hence how it reacts to host transitions. Immediately following a transition to a new niche after a host switch, resources will be abundant and mean fitness will be low. As such, in this period where cells grow and adapt to the new niche, cell death with be driven by fitness, with the effect of diminishing the less fit genotypes via natural selection. However, once the maximal fitness for this population in this niche has been reached, the cell fate will be determined by resource availability which is entirely random and unrelated to fitness. As such, when the population hits its carrying capacity, in this case enacted by the availability of resources, the fitness of the population ceases to increase in any meaningful capacity. Crucially, this *helps maintain genetic variance in the population*, severely slowing convergence to the optimal genotype, and so allows alleles which may be less fit to survive until the next host

Figure 1: Schematic plot showing how the proportion of cell death between fitness and resource related death changes with starting population size in a single generation. Dashed lines represent fitness-related death and solid lines represent resource-related death and different colours correspond to fixed fitness values as indicated in the legend. The carrying capacity in this case is 2.5 million

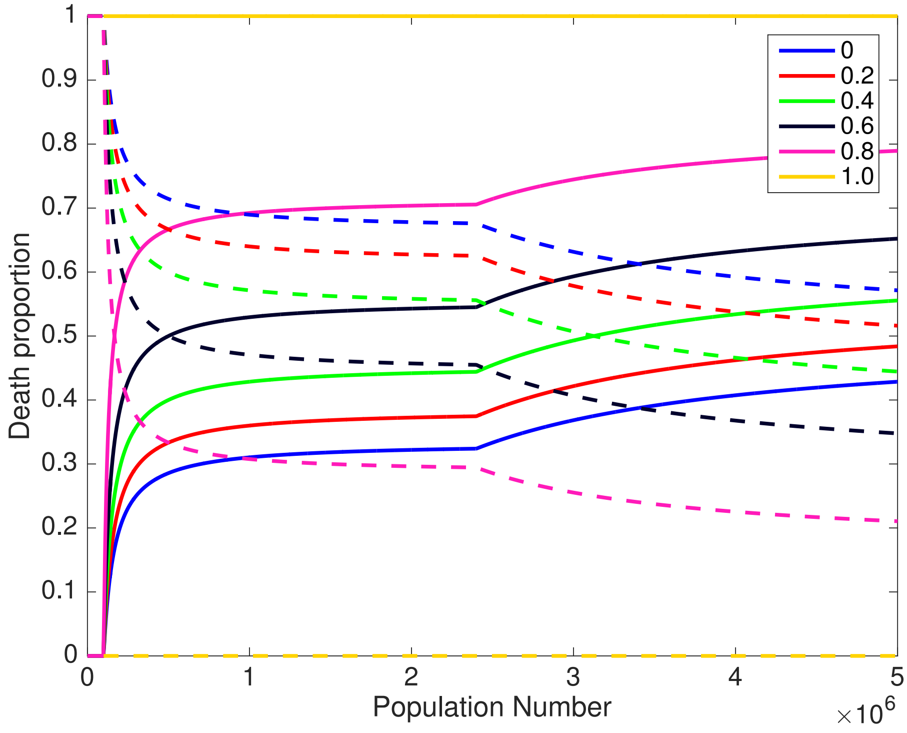

transition, where they may have a higher fitness. For populations above the carrying capacity, the likelihood of any death being due to resources becomes even more likely, which can be seen in the change in the curves after 2.5 million.

This reenforces the importance of recombination rate to the dynamics and long term viability of the population. As the rate of convergence to the optimal genotype increases with increasing recombination rate, when the recombination rate and carrying capacity are high, the population will have converged to an optimal genotype before the carrying capacity is reached. This has the

knock on effect of purging more alleles from the population, and reducing variance, potentially leading to poor adaptation in subsequent niches. Conversely, if the recombination rate is so low that the carrying capacity is reached before the population is sufficiently adapted, then alleles are lost at random meaning the population may become ill equipped to survive in either niche. As such, this gives more insight into why, in multiple hosts, an intermediate recombination rate is favoured as the interplay with the carrying capacity provides a trade-off between adaptation and maintenance of currently unfit alleles.

3 References

[1] Kauffman, S and Weinberger, E. The NK Model of rugged fitness landscapes and its application to the maturation of the immune response. J Theor Bio. 1989, 141 (2): 211245.

[2] Maiden MC, Bygraves JA, Feil E, Morelli G, Russell JE, Urwin R, Zhang Q, Zhou J, Zurth K, Caugant DA, Feavers IM, Achtman M and Spratt BG. Multilocus sequence typing: a portable approach to the identification of clones within populations of pathogenic microorganisms. Proc Natl Acad Sci. 1998, 95(6): 3140-3145.
